# A single determination of C-reactive protein does not suffice to declare a patient with a diagnosis of axial spondyloarthritis ‘CRP-negative’

**DOI:** 10.1186/s13075-018-1707-8

**Published:** 2018-09-14

**Authors:** Robert Landewé, Tommi Nurminen, Owen Davies, Dominique Baeten

**Affiliations:** 1 Clinical Immunology and Rheumatology, Amsterdam Rheumatology & Immunology Center and Academic Medical Center, University of Amsterdam, Amsterdam, Netherlands; 2 UCB Pharma, Monheim, Germany; 3 UCB Pharma, Slough, UK; 4 UCB Pharma, Brussels, Belgium

**Keywords:** C-reactive protein, Non-radiographic axial spondyloarthritis, Inflammation

## Abstract

**Background:**

To be eligible to receive treatment with an anti-tumour necrosis factor (TNF), non-radiographic axial spondyloarthritis (nr-axSpA) patients require either elevated levels of C-reactive protein (CRP) (CRP > upper limit of normal (ULN)) or magnetic resonance imaging assessment showing inflammation of the sacroiliac joints, in addition to meeting criteria for high disease activity. Many axSpA patients are classified as ‘CRP-negative’, or CRP normal, despite having levels close to the ULN, and are therefore formally ineligible for treatment. The aim of this study was to investigate the likelihood of a CRP test indicating elevated levels in axSpA patients that have previously tested CRP normal.

**Methods:**

RAPID-axSpA (NCT01087762) enrolled patients who were either magnetic resonance imaging positive or had elevated CRP (> ULN: 7.9 mg/L). CRP data from the double-blind period for placebo-randomised patients until re-randomisation to certolizumab pegol (week 16 for ASAS20 non-responders/week 24 for ASAS20 responders) were analysed. CRP was assessed at screening, baseline, and nine time points to week 24. Linear mixed models were used to investigate time trends, variability, and correlations of CRP data.

**Results:**

Of 106 placebo-randomised patients with baseline CRP assessments, 26 (25%) tested CRP normal at baseline, of whom 13 (50%) had ≥ 1 test indicating elevated CRP to week 16. Of 80/106 (75%) patients with elevated baseline CRP, 25 (31%) had ≥ 1 normal CRP test to week 16. Linear mixed models did not reveal changes in mean CRP across placebo patients from baseline to week 24.

**Conclusions:**

In axSpA patients with CRP < ULN the CRP test should be repeated after ≥ 4 weeks as there is a substantial chance of finding a positive result for elevated CRP at subsequent testing, thereby allowing the patient access to treatment.

**Trial registration:**

ClinicalTrials.gov, NCT01087762. Registered on 16 March 2010.

**Electronic supplementary material:**

The online version of this article (10.1186/s13075-018-1707-8) contains supplementary material, which is available to authorized users.

## Background

Axial spondyloarthritis (axSpA) is a chronic inflammatory disease involving the spine and/or sacroiliac joints. Typically, axSpA patients are further classified as having either ankylosing spondylitis (AS) or non-radiographic (nr)-axSpA, depending on the presence or absence of radiographic sacroiliitis, respectively [1]. It is generally accepted that these two subpopulations represent a spectrum of the same disease, and both subgroups have been shown to experience a similar burden of disease and similar clinical response to anti-tumour necrosis factor (TNF) therapy [2, 3]. Nevertheless, concerns have been voiced regarding the possibility of spontaneous remission in nr-axSpA patients, and potential overtreatment with biologics [4]. In the US, anti-TNFs have received Food and Drug Administration (FDA) approval for the treatment of AS [5–8], but are yet to obtain approval for the treatment of nr-axSpA [9]. In contrast, four anti-TNFs have been approved by European regulatory authorities for the treatment of patients with active nr-axSpA on the condition that they must exhibit objective signs of inflammation, demonstrated by magnetic resonance imaging (MRI) and/or elevated levels of C-reactive protein (CRP) [10–12].

The CRP level in axSpA patients is a key indicator of inflammation and is also considered to be a predictor of clinical response to anti-TNF treatment. In a number of studies, patients with elevated CRP at baseline have demonstrated significantly greater responses to anti-TNFs compared with patients with low CRP levels as well as those receiving placebo treatment [13, 14].

To be eligible to receive treatment with an anti-TNF in Europe, Assessment of SpondyloArthritis international Society (ASAS) treatment recommendations state that nr-axSpA patients must have either a positive CRP assessment (elevated CRP greater than the upper limit of normal (ULN)) or a positive MRI showing inflammation of the sacroiliac joints, in addition to meeting criteria for high disease activity [15, 16]. Many axSpA patients with normal CRP levels are classified as CRP ‘negative’ despite having levels close to the ULN, and are therefore formally ineligible for treatment. The natural degree of CRP fluctuation in patients who have not received anti-TNF therapy is not well understood [17], and should be investigated further.

In this post-hoc analysis of data from a phase 3 randomised controlled trial, we evaluated the consistency of CRP testing in patients with active axSpA.

## Methods

### Study design

The phase 3 RAPID-axSpA study (NCT01087762) evaluated the efficacy and safety of certolizumab pegol (CZP) in patients with active axSpA. The trial was double-blind and placebo-controlled to week 24, dose-blind to week 48 and open-label to week 204. Data here are taken from the initial 24-week, double-blind, placebo-controlled period.

At week 0, patients were randomised 1:1:1 to placebo, CZP 400 mg every 4 weeks (Q4W), or CZP 200 mg every other week (Q2W). Patients randomised to placebo who did not meet ASAS 20% response criteria (ASAS20) at either weeks 14 or 16 were allocated to escape treatment (re-randomised 1:1 to either CZP 200 mg Q2W or CZP 400 mg Q4W). Placebo-randomised patients who did achieve an ASAS20 response were re-randomised to CZP at week 24 (the end of the double-blind period).

We analysed data only from placebo-randomised patients until their point of re-randomisation to CZP at either week 16 or week 24.

The trial enrolled patients with a clinical diagnosis of axSpA, fulfilling ASAS criteria, with active disease defined by each of the Bath Ankylosing Spondylitis Disease Activity Index (BASDAI) score ≥ 4, spinal pain ≥ 4 on a 0–10 numeric rating scale, CRP > ULN (7.9 mg/L) and/or sacroiliitis on MRI as defined by ASAS classification criteria, within 3 months prior to screening. One re-testing of subjects who failed screening due to the CRP level was permitted. All patients had an inadequate response to, or intolerance of, ≥ 1 non-steroidal anti-inflammatory drug (NSAID). Conventional background medications at stable dose levels were allowed during the screening and double-blind periods of the study. Detailed inclusion and exclusion criteria have been previously presented [18]. Approval from the Independent Ethics Committee or Institutional Review Board was obtained. Informed consent according to the Declaration of Helsinki was collected from all patients.

### Study procedures and evaluations

The primary outcome (ASAS20 responder rate at week 12) [18], as well as safety, efficacy, and patient-reported outcomes to week 204, have been reported previously [19]. CRP level was assessed at screening, baseline, and at nine time points (weeks 1, 2, 4, 8, 12, 16, 18, 20, and 24) to week 24. Serum samples were analysed in two central laboratories: the European central laboratory (~ 60% of samples) used a conventional CRP assay with a lower limit of quantification (LLQ) of 3 mg/L, while the samples from North and Latin America (~ 40% of samples) were analysed with a high-sensitivity assay (LLQ 0.1 mg/L). Elevated CRP was defined as CRP > ULN (7.9 mg/L) and values ≤ 7.9 mg/L were considered to have normal CRP levels.

### Statistical analysis

Linear mixed models (LMM) were used to investigate time trends, variability, and correlations of CRP data following a Ln(CRP mg/L + 1) transformation [20]. LMM analyses at the group level were used to evaluate possible placebo response, whilst LMM analyses at the patient level allowed auto-correlations (i.e. the similarity of two results depending on the time interval between them) to be quantified. Based upon the results from LMM analyses, simplified descriptive summaries were generated to illustrate reproducibility of CRP tests. Since valid post-week 16 evaluations were available from ASAS20 responders, certain descriptive analyses were restricted up to week 16 a priori to avoid data confoundment. For CRP values below the lower limit of quantification, half the lower limit was used for analysis. Statistical analyses were performed using SAS® version 9.3 (SAS Institute, Cary, NC, USA).

## Results

### Patient disposition and baseline characteristics

In RAPID-axSpA, a total of 325 patients were randomised to treatment at week 0, 107 to placebo, and 218 to CZP. Of 106 patients receiving placebo with baseline CRP assessments, 26 (25%) had normal CRP tests and 80 (75%) had elevated CRP levels.

### Is a patient’s CRP status stable?

Of the 26 patients with normal CRP levels at baseline, 13 (50%) subsequently had at least one test indicating elevated CRP levels to week 16, and 1 (4%) patient had elevated CRP for all following assessments to week 16. For the 80/106 patients with elevated CRP at baseline, 25 (31%) subsequently had at least one CRP test with normal CRP levels. No patients exhibited normal CRP levels for all subsequent assessments to week 16 (Table 1).

**Table 1:** Likelihood of finding elevated C-reactive protein (CRP) tests to week 16 after initial tests at baseline

Proportion of subsequent CRP tests elevated	Baseline CRP test normal (*n* = 26)	Baseline CRP test elevated (*n* = 80)
0%	13 (50%)	0 (0%)
> 0–20%	1 (4%)	1 (1%)
> 20–40%	4 (15%)	3 (4%)
> 40–60%	4 (15%)	11 (14%)
> 60–80%	1 (4%)	6 (8%)
> 80–100%	2 (8%)	4 (5%)
100%	1 (4%)	55 (69%)

Data included are from weeks 0, 1, 2, 4, 8, 12, and 16. Elevated CRP was defined as CRP > upper limit of normal (7.9 mg/L) and values ≤ 7.9 mg/L were considered normal

### How does CRP vary over time? Results from LMM analyses

There was no strong evidence of change in mean CRP from baseline to week 24 (note that all patients included were randomised to placebo). However, variation in CRP level was observed over time in individual patients. Analysis of within-subject auto-correlation indicated that short-term assessments (1–2 weeks apart) were strongly correlated, i.e. provided similar results. This correlation decreased in assessments separated by periods of ≥ 4 weeks. Results from LMM analyses are presented in Additional file 1.

### Reproducibility of CRP status: what is the likelihood of a change in CRP status at a future assessment?

**Fig. 1:**
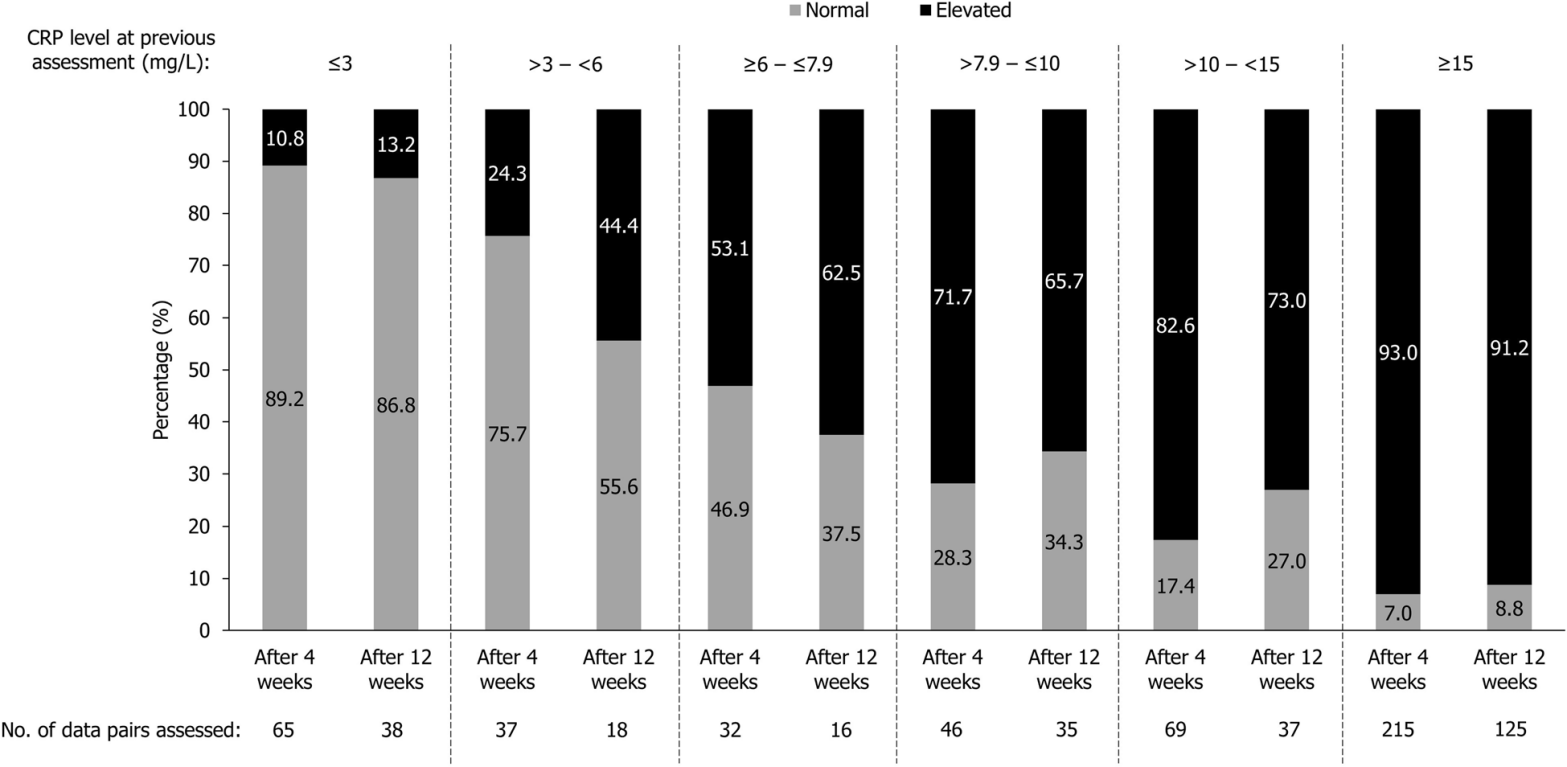
Percentage of C-reactive protein (CRP) tests subsequently elevated or normal by previous CRP level. Based on all available within-patient pairs of CRP assessment 4 or 12 weeks apart. Data included are from weeks 0, 4, 8, 12, 16, 20, and 24. Elevated CRP was defined as CRP > ULN (7.9 mg/L) and values ≤ 7.9 mg/L were considered normal

Given the observed lack of systematic group-level trends in CRP, we calculated how often a value within a given range was followed by an elevated CRP test result at a subsequent assessment (assuming that any available CRP assessments could represent a placebo-randomised patient’s first and only available result). Descriptive evaluations demonstrated that 10.8% (*n* = 7) of ≤ 3 mg/L CRP assessments would result in elevated CRP levels (defined as > 7.9 mg/L) 4 weeks following their initial assessment (Fig. 1). This increased to 53.1% (*n* = 17) when the original CRP assessment was between ≥ 6 and ≤ 7.9 mg/L. Similarly, 7.0% (*n* = 15) of CRP assessments ≥ 15 mg/L tested normal after 4 weeks, which increased to 28.3% (*n* = 13) when the initial CRP test was between > 7.9 and ≤ 10 mg/L. If assessments were repeated 12 weeks later, 62.5% (*n* = 10) of CRP tests between ≥ 6 and ≤ 7.9 mg/L would be followed by an elevated CRP. For patients with a CRP level between > 7.9 and ≤ 10 mg/L, the likelihood of having normal CRP levels increased to 34.3% (*n* = 12) after 12 weeks.

## Discussion

The results of this study showed that, despite the patient-reported improvements in disease activity in patients treated with placebo [18], CRP levels (as an indicator of inflammation) were not affected. However, fluctuation in CRP levels at the patient level were common: 50% of patients who exhibited normal CRP levels at baseline exhibited at least one elevated CRP result within the following 16 weeks, and 31% changed from elevated to normal CRP levels. In addition, further model-based and descriptive analyses demonstrated that patients with low CRP values at their first evaluation had a realistic chance of exhibiting elevated CRP upon re-evaluation. These results are in agreement with those from the ABILITY-1 study [21], in which 24.6% (14/57) of placebo-treated patients with normal CRP levels at baseline developed elevated CRP levels after 12 weeks, suggesting that a change in a patient’s CRP status is more common than previously thought.

Alongside clinical parameters, assessing the likelihood of fluctuation in CRP levels could help inform treatment decisions for nr-axSpA patients. This would be particularly important for those with sacroiliitis on MRI and normal CRP, but high disease activity, suggesting a potential benefit from anti-TNF therapy. Introducing a further CRP test at least 4 weeks after the initial test (due to more short-term test results being highly auto-correlated) could assist with the more accurate categorisation of patient CRP profiles. Furthermore, the likelihood of demonstrating elevated CRP at a later time point is higher in patients with CRP levels just below the ULN.

After 4 weeks, auto-correlation continued to decrease to week 12. Subsequently, an additional assessment after 4 or 12 weeks could theoretically be acceptable, although recommendations should not be made based on these correlation data alone. It may be appropriate to distinguish between these two time points using practical considerations; an assessment at week 4 could be used in cases where rapid initiation of biological therapy is required, whereas week 12 may be more suitable if this time point coincides with a patient’s next clinical assessment.

One of the limitations of these post-hoc analyses was that the sample size of the patient population used was relatively small; therefore, the interpretation of these results should be treated with caution. A further limitation was that all patients enrolled in the trial were required to have elevated CRP levels or active inflammation on MRI at screening; patients with elevated CRP at screening would be more likely to have elevated CRP at a later time point. Additionally, a relatively high threshold of ULN (> 7.9 mg/L) was used; different levels of variation may be seen at a lower threshold. Furthermore, the hypothesis that patients with clinically active disease but normal CRP levels at baseline should be re-tested is only relevant for those patients who do not have active inflammation on MRI at baseline. However, all patients in RAPID-axSpA with normal CRP values had demonstrated sacroiliitis on MRI at baseline; therefore, it is unclear whether CRP values in patients with active nr-axSpA who do not show active inflammation on MRI behave in a same manner. To make an evidence-based recommendation regarding the re-testing of CRP levels, it is recommended that additional data are collected to explore whether a patient with clinically active nr-axSpA (without sacroiliitis on MRI) with normal CRP at baseline and elevated CRP levels at a later time point is likely to respond to anti-TNF therapy.

## Conclusions

For nr-axSpA patients with no signs of inflammation on MRI and normal CRP levels, in addition to a high disease activity indicative of a potential benefit from anti-TNF treatment, the CRP test should be repeated after at least 4 weeks. There is a substantial chance of finding elevated CRP levels upon subsequent testing, thus making the patient eligible for treatment options such as anti-TNF therapies.

## Key messages

- Non-radiographic axSpA patients must exhibit inflammation (via MRI/CRP) to access anti-TNF treatment.
- Non-radiographic axSpA patients with normal CRP levels have a substantial chance of demonstrating elevated CRP at subsequent re-test.
- In non-radiographic axSpA patients, the CRP test should be repeated after at least 4 weeks.

## Additional file

Additional file 1: Linear Mixed-effects Modelling: Additional Information. (DOCX 59 kb)

## Abbreviations

AS: Ankylosing spondylitis
ASAS: Assessment of SpondyloArthritis international Society
axSpA: Axial spondyloarthritis
BASDAI: Bath Ankylosing Spondylitis Disease Activity Index
CRP: C-reactive protein
CZP: Certolizumab pegol
FDA: Food and Drug Administration
LLQ: Lower limit of quantification
LMM: Linear mixed models
MRI: Magnetic resonance imaging
nr: Non-radiographic
NSAID: Non-steroidal anti-inflammatory drug
Q2W: Every 2 weeks
Q4W: Every 4 weeks
TNF: Tumour necrosis factor
ULN: Upper limit of normal
