# Supplementary material for: A single determination of C-reactive protein does not suffice to declare a patient with a diagnosis of axial spondyloarthritis ‘CRP-negative’
Source: Arthritis Res Ther. 2018 Sep 14;20:209. doi: 10.1186/s13075-018-1707-8 (PMC6137888; doi:10.1186/s13075-018-1707-8)
Supplement: Supplementary file 1 — Linear Mixed-effects Modelling: Additional Information. (DOCX 59 kb) [file 13075_2018_1707_MOESM1_ESM.docx]

**Additional file 1.** Linear Mixed-effects Modelling Objectives

Linear mixed-effects models (LMM) were fitted to Ln(CRP mg/L + 1) data in order to:

1. Evaluate differences in average CRP levels between time points, including a test for linear trends over time.
   - Stability of average CRP was considered a prerequisite for the validity of any subsequent analyses: if relevant changes in average CRP levels were observed, such as a decrease following inception of placebo treatment, then the data could not be considered to represent ‘natural’ variability of CRP. It was assumed that no systematic changes affected CRP data.
2. Identify the minimum number of covariance parameters that sufficiently characterised the variability and within-patient correlations in the data.
3. Estimate correlations between assessments within the same patient, and their dependence on the length of time interval between those assessments. Within-patient correlation reduction over time impacts:
   - the interpretation of analyses where all post-baseline assessments are combined.
   - the length of time after which a CRP assessment should be repeated for a patient in clinical practice.

***Model Fitting and Results***

LMMs were fitted with ‘visit’ as a categorical fixed factor (i.e. allowing for a different average CRP at each visit). The covariance models that were fitted and compared based on the finite-sample corrected version of Akaike’s information criterion (AICc) [1] are presented in **Table S1**.

**Table S1.** Fitted covariance models

| **Model** | **Random effects** | **Residual covariance** | **Covariance parameters (n)** | **AICc** |
| --- | --- | --- | --- | --- |
| 1 | - | Unstructured | 55 | 1467.0 |
| 2 | Intercept | Independent residuals | 2 | 1552.1 |
| 3 | Intercept | Exponential autocorrelation | 4 | 1467.6 |
| 4 | Intercept | Gaussian autocorrelation | 4 | 1470.4 |
| 5 | Intercept, Time | Independent residuals | 4 | 1490.1 |
| 6 | Intercept, Time | Exponential autocorrelation | 6 | 1467.3 |
| 7 | Intercept, Time | Gaussian autocorrelation | 6 | 1464.4 |

AICc: Akaike’s information criterion.

The covariance structure that fitted the data most conclusively was Model 7. Each patient had a unique linear regression line describing the level (random intercept) and linear trajectory of change over time (random slope). In addition to these random effects, Gaussian residual autocorrelation was required to account for similarity of assessments at short intervals. It could be deduced from the covariance parameter estimates that, among all assessments (n=829), repeated assessments of the same patients were correlated (i.e. similar) and this correlation declined over time (0.89, 0.78 and 0.57 between repeated assessment at intervals of 1, 4 and 24 weeks, respectively). This covariance model was applied when testing differences of average CRP levels between visits. There was no evidence of changes in average CRP level in Type III tests or parameter contrast testing for linear trends over time (p=0.439 and p=0.485, respectively).

***LMM Conclusions***

Given the lack of difference observed in average CRP between visits, it can be concluded that the clinical trial setting and placebo treatment did not induce changes in CRP levels. Thereby, the observed data patterns can be assumed to represent ‘natural’ variability in CRP levels in the target patient population. The decrease in between-assessment correlation over time suggests that reproducibility of normal CRP tests is dependent on the length of time between the first and second assessments. Therefore, as described in the main text, reproducibility of CRP tests was analysed descriptively for two distinct scenarios: (1) for a CRP test repeated after 4 weeks and (2) for a CRP test repeated after 12 weeks. Reproducibility was analysed by considering all pairs of CRP assessments from the same individual (4 or 12 weeks apart) and then evaluating how frequently the later of the two assessments indicated elevated CRP, depending on the CRP level in the preceding assessment.

***Reference***
1. Burnham KP, Anderson DR. Model Selection and Inference: A Practical Information-Theoretic Approach. New York: Springer-Verlag; 1998.
